# Dexmedetomidine only regimen for long-term sedation is associated with reduced vasopressor requirements in septic shock patients: A retrospective cohort study from MIMIC-IV database

**DOI:** 10.3389/fmed.2023.1107251

**Published:** 2023-02-27

**Authors:** Lulan Li, Xiaotong Shi, Ming Xiong, Karen Kong, Zhongqing Chen, Shiyu Zhou, Zhenhua Zeng, Shengli An, Bo Xu

**Affiliations:** ^1^Department of Anesthesiology, General Hospital of Southern Theatre Command of PLA, The First School of Clinical Medicine, Southern Medical University, Guangzhou, China; ^2^Department of Critical Care Medicine, Nanfang Hospital, Southern Medical University, Guangzhou, China; ^3^Department of Biostatistics, School of Public Health (Guangdong Provincial Key Laboratory of Tropical Disease Research), Southern Medical University, Guangzhou, Guangdong, China; ^4^Department of Anesthesiology & Peri-Operative Medicine, New Jersey Medical School, Rutgers, United States

**Keywords:** dexmedetomidine, norepinephrine, septic shock, ICU, sedation

## Abstract

**Background:**

Previous studies have shown that dexmedetomidine (DEX) may be associated with reduced vasopressor requirements in septic shock patients, however, long-term DEX-only sedation in reducing vasopressor requirements is still controversial.

**Methods:**

A retrospective study was conducted among patients with septic shock on mechanical ventilation using the Medical Information Mart for Intensive Care IV (MIMIC-IV) database. The primary outcome was the ratio of norepinephrine equivalent dose to mean arterial pressure (NEq/MAP) in the first 72 h after DEX or other sedatives for sedation. The secondary outcomes were key organ function parameters, 28-day mortality, and 90-day mortality. Univariate, propensity score matching (PSM), and generalized linear mixed model (GLMM) analyses were performed.

**Results:**

DEX was associated with decreased NEq/MAP in the first 72 h (difference = 0.05, 95% CI = –0.02–0.08, *p* = 0.002) after adjusting for confounders in the GLMM analysis. The DEX group was also associated with a lower heart rate, cardiac output (CO), lactate level, aspartate transaminase (AST) level, and higher PaO_2_/FiO_2_ ratio (*p* < 0.0125). Moreover, DEX only sedation was associated with reduced 90-day mortality (OR = 0.60, 95% CI = 0.37–0.94, *p* = 0.030).

**Conclusion:**

DEX may be associated with decreased vasopressor requirements, improved AST and PaO_2_/FiO_2_ levels, and reduced 90-day mortality in patients with septic shock, which warrants further study.

## Introduction

Septic shock refers to sepsis with hypotension and blood lactate level of >2.0 mmol/l that cannot be corrected by positive fluid therapy (1). Current epidemiological data have shown an increased incidence of septic shock and a mortality rate ranging from 26 to 42% (2, 3). Vasopressors are the cornerstones of shock treatment (4). Specifically, norepinephrine (NE) is used as first-line vasopressor therapy (5). Theoretically, the sympathetic nervous system (SNS) plays a vital role in septic shock by enabling patients to maintain smooth muscle cell contraction (6). However, prolonged activation of the SNS in septic shock patients may elevate levels of circulating catecholamines (7), causing down-regulation and desensitization of α-adrenergic receptors on the surface of smooth muscle cells (8). This can make patients hyposensitive or insensitive to exogenous catecholamines, leading to deterioration due to refractory shock (9). There is an urgent need to reduce vasopressor requirements because some studies have revealed that the need for NE is one of the indicators of severity in patients with septic shock (10–12).

Data from animal studies have shown that dexmedetomidine (DEX) may reduce NE requirements in septic shock settings (13, 14). Recent clinical studies have also revealed that DEX for sedation may lower vasopressor requirements in septic shock patients, among them, the sample size is relatively small and the observation time is relatively short (15–18). A crossover trial included only 38 patients and was observed for 12 h, showing a decrease in NE doses 4 h after DEX only administration (17), however, the duration of DEX infusion was limited to 4 h. A retrospective study including 83 patients found that DEX was associated with lower vasopressor requirements to maintain target MAP in the first 48 h (15), however, this study could not eliminate the confounding effects of other sedatives (19). Moreover, a randomized trial recruited 66 patients and found that DEX- only sedation tended to reduce NE compared to normal saline (16). Inconsistent with the above three studies, a comparative study enrolled a small sample size of 24 patients and reported that sedation with DEX + propofol required a similar amount of NE as midazolam + propofol (18), and the relationship between DEX and NE in this study could not eliminate the confounding effects of propofol. It is important to note that the time window for reversing shock and reducing NE is also very important, nearly half of the deaths attributable to septic shock occur within the first 72 h (20, 21). However, previous studies have reported that DEX sedation for more than 24 h may cause withdrawal syndrome with sudden cessation (22, 23), thus the long-term effects as long as 72 h of DEX-only sedation in septic shock patients remain unclear. Herein, we investigated the long-term use of a DEX-only sedation regimen for hemodynamic changes, especially vasopressor requirements in septic shock patients, using a large sample size from the public database Medical Information Mart for Intensive Care IV (MIMIC-IV).

## Materials and methods

We utilized the MIMIC-IV critical care database, which includes ICU patient data from 2008 to 2019. MIMIC-IV was established by the Massachusetts Institute of Technology (Cambridge, MA) and Beth Israel Deaconess Medical Center (Boston, MA), and all patient data were collected with IRB approval. Author Zhou was responsible for data extraction from the database (certification number 35931520).

### Inclusion and exclusion criteria

Adult (age ≥ 18 years) septic shock patients on mechanical ventilation who received vasopressors before sedation were included in our study. The diagnosis of septic shock was based on the ICD-10 codes in the MIMIC-IV database. The exclusion criteria were readmission to ICU, length of stay in ICU was less than 24 h, received oral alpha-agonist clonidine, received DEX outside ICU stay.

### Study design

This was a single-center, retrospective cohort study. Selected patients were separated into two groups, DEX and non-DEX, based on whether they were sedated with DEX or other sedatives. The DEX group included patients who only received DEX for sedation, and the non-DEX group included patients who received propofol, or midazolam for sedation. Our study did not consider patients who received both DEX and propofol or midazolam. We collected demographic characteristics of patients at ICU admission including age, sex, and ethnicity; baseline clinical data, including respiratory rate, heart rate, temperature, mean arterial pressure (MAP), oxygen saturation (SpO_2_), white blood cells (WBCs), platelet count, lactate level, serum creatinine level, urinary output within 24 h after ICU admission, oxygenation index (PaO_2_/FiO_2_), infection sites, incidence, and severity of acute kidney injury (AKI), and acute respiratory distress syndrome (ARDS), use of analgesic drugs, mainly morphine and fentanyl, and drug histories, such as diltiazem and other anti-hypertensive drugs. Richmond Agitation Sedation Scale (RASS), Critical Care Pain Observation Tool (CPOT), Simplified Acute Physiology II (SAPS II) score, sequential organ failure assessment (SOFA) score, Charlson comorbidity index (CCI) score, Glasgow Coma Scale (GCS); and parameters reflecting various organ functions and follow-up data for up to 90 days for all eligible patients were used.

### Clinical outcomes

The primary outcome was the ratio of norepinephrine equivalent dose (NEq) to MAP (NEq/MAP) in the first 72 h after receiving DEX or other sedatives. The average vasopressor dose was expressed as NEq, which was calculated as norepinephrine + epinephrine + vasopressin/0.4 based on previous studies (15, 24) as a measure of vasopressor dose in the first 72 h. MAP was calculated by DBP +1/3 (SBP-DBP). The NEq/MAP ratio was analyzed instead of just NEq to account for differences in target MAPs among patients, because the target MAP in different conditions might differ and vasopressor drugs other than NE would also be used to maintain blood pressure. Secondary outcomes included other hemodynamic parameters, such as heart rate (HR), cardiac output (CO), lactate level, and parameters that reflect the function of multiple organs: serum creatinine level, daily urine output, alanine transaminase (ALT), aspartate transaminase (AST), total bilirubin (TBIL), and oxygenation index (PaO_2_/FiO_2_). Patient outcomes including 28-day and 90-day mortality, were also analyzed. The CPOT and RASS scores were compared between the two groups to assess differences in the levels of sedation and analgesia.

### Statistical analysis

In the baseline analysis, continuous variables were shown as mean and standard deviation (SD) or median and interquartile range (IQR) as appropriate. The DEX and non-DEX groups were compared using independent t-test or Mann–Whitney U test. Categorical variables are shown as numbers and percentages (%) and were compared using the chi-square tests or Fisher’s exact test. Missing values were inferred using the assumption of missing at random (MAR), and multivariate imputation by chained equation (MICE) methods was used to perform multiple imputations.

Propensity score matching (PSM) was performed to balance the confounding factors. Variables, including all the variables in Table 1, were chosen to generate the PS based on clinical significance and previous studies. The propensity score was calculated using logistic regression. Matching was performed using the nearest neighbor method, with a caliper value limited to 0.2. Match quality was determined using standardized mean differences (SMDs). Subsequent analyses, such as NEq/MAP in the first 72 h, mortality rates and biochemical markers, were all based on the data after PSM.

**Table 1:** Baseline characteristics in DEX group and non-DEX group before and after propensity score matching (PSM).

Characteristics	Before PSM			After PSM		
	DEX	Non-DEX	*p*	DEX	Non-DEX	*p*
	(*n* = 221)	(*n* = 1,424)		(*n* = 215)	(*n* = 215)	
Age(year), median (IQR)	64.31 [54.50, 74.17]	67.65 [56.39, 78.80]	0.004	65.03 [54.56, 74.87]	66.26 [54.98, 76.82]	0.336
Sex, male (%)	132 (59.7)	780 (54.8)	0.192	129 (60.0)	129 (60.0)	1
Ethnicity, non-white (%)	92 (41.6)	601 (42.2)	0.93	92 (42.8)	95 (44.2)	0.846
Married (%)	75 (33.9)	560 (39.3)	0.145	75 (34.9)	83 (38.6)	0.484
Insurance (%)	110 (49.8)	815 (57.2)	0.045	107 (49.8)	114 (53.0)	0.563
Admission type, emergency (%)	104 (47.1)	836 (58.7)	0.001	104 (48.4)	102 (47.4)	0.923
Weight (kg), median (IQR)	82.60 [68.00, 99.10]	81.00 [67.00, 98.90]	0.713	104 (48.4)	102 (47.4)	0.923
Respiratory rate (/min), median (IQR)	22.00 [19.35, 25.30]	21.43 [18.54, 24.73]	0.085	21.98 [19.32, 25.15]	22.43 [18.97, 24.81]	0.89
Heart rate (bpm), median (IQR)	90.82 [81.23, 105.40]	94.23 [80.22, 107.18]	0.25	90.89 [81.26, 105.40]	92.40 [79.98, 105.50]	0.752
Temperature (°C), median (IQR)	37.03 [36.73, 37.40]	36.96 [36.59, 37.43]	0.043	37.03 [36.72, 37.40]	37.12 [36.68, 37.60]	0.234
MAP (mmHg), median (IQR)	75.82 [71.47, 80.44]	73.73 [69.72, 78.21]	<0.001	75.74 [71.45, 80.29]	75.56 [70.92, 80.47]	0.869
SpO_2_ (%), median (IQR)	96.89 [95.52, 98.24]	97.10 [95.38, 98.52]	0.59	96.89 [95.54, 98.22]	97.12 [95.48, 98.57]	0.54
WBCs (×10^9^), median (IQR)	14.10 [10.36, 19.88]	14.01 [9.14, 19.86]	0.622	14.22 [10.43, 19.94]	14.00 [9.26, 19.62]	0.479
Platelet (×10^12^), median (IQR)	170.00 [109.50, 246.40]	172.83 [109.56, 246.00]	0.721	170.00 [108.44, 245.03]	167.67 [106.50, 244.63]	0.99
Lactate (mmol/L), median (IQR)	2.20 [1.48, 3.32]	2.46 [1.66, 4.14]	0.002	2.20 [1.49, 3.31]	2.15 [1.60, 3.33]	0.876
Serum creatinine (mg/dL), (median [IQR])	1.50 [0.95, 2.43]	1.60 [1.02, 2.53]	0.612	1.50 [0.96, 2.39]	1.45 [1.01, 2.40]	0.975
Urine output within the first 24 h (L), median (IQR)	0.98 [0.48, 1.79]	0.99 [0.45, 1.71]	0.301	0.26 [0.05, 0.96]	0.19 [0.05, 1.09]	0.92
PaO_2_/FiO_2_ (mmHg), median (IQR)	151.00 [87.50, 260.00]]	140.00 [77.00, 246.43	0.138	150.00 [91.00, 245.83]	142.00 [78.00, 248.57]	0.61
SAPS II score,median (IQR)	47.00 [38.00, 58.00]	51.00 [42.00, 62.00]	0.002	48.00 [38.00, 59.00]	48.00 [39.50, 58.00]	0.871
SOFA score, median (IQR)	12.00 [9.00, 14.00]	12.00 [9.00, 14.00]	0.812	12.00 [9.00, 14.00]	12.00 [10.00, 14.00]	0.923
CCI score, median (IQR)	6.00 [4.00, 8.00]	6.00 [4.00, 8.00]	0.161	6.00 [4.00, 8.00]	6.00 [4.00, 8.00]	0.816
GCS score, median (IQR)	10.00 [6.00, 13.00]	10.00 [4.00, 14.00]	0.211	10.00 [6.00, 13.00]	9.00 [4.00, 13.00]	0.717
Infection side, n (%)						
Blood	200 (90.5)	1,203 (84.5)	0.025	194 (90.2)	188 (87.4)	0.444
Respiratory	89 (40.3)	430 (30.2)	0.003	85 (39.5)	89 (41.4)	0.768
Gastrointestinal	15 (6.8)	68 (4.8)	0.269	14 (6.5)	17 (7.9)	0.709
Skin/soft tissues	3 (1.4)	34 (2.4)	0.473	3 (1.4)	2 (0.9)	1
Urinary	105 (47.5)	540 (37.9)	0.008	101 (47.0)	102 (47.4)	1
AKI stage (%)			0.459			0.961
3	3 (1.4)	19 (1.3)		3 (1.4)	4 (1.9)	
2	5 (2.3)	16 (1.1)		4 (1.9)	5 (2.3)	
1	14 (6.3)	73 (5.1)		13 (6.0)	12 (5.6)	
0	199 (90.0)	1,316 (92.4)		195 (90.7)	194 (90.2)	
ARDS (%)			0.83			0.422
Severe	41 (22.5)	239 (21.6)		41 (22.9)	33 (19.4)	
Moderate	75 (41.2)	483 (43.6)		74 (41.3)	82 (48.2)	
Mild	66 (36.3)	385 (34.8)		64 (35.8)	55 (32.4)	
Surgery (%)	43 (19.5)	312 (21.9)	0.461	43 (20.0)	40 (18.6)	0.807
Diltiazem (%)	26 (11.8)	197 (13.8)	0.465	26 (12.1)	22 (10.2)	0.646
Hypertension	85 (38.5)	519 (36.4)	0.615	82 (38.1)	81 (37.7)	1
Anti-HTN	19 (8.6)	119 (8.4)	1	18 (8.4)	13 (6.0)	0.456
Fentanyl (%)	156 (70.6)	997 (70.0)	0.925	151 (70.2)	153 (71.2)	0.916
Morphine (%)	56 (25.3)	402 (28.2)	0.417	55 (25.6)	58 (27.0)	0.827
Tachyarrhythmias	193 (87.3)	1,146 (80.5)	0.019	187 (87.0)	186 (86.5)	1
Doses of NEq (ug/kg/min) median (IQR)	0.10 [0.05, 0.20]	0.10 [0.05, 0.25]	0.025	0.10 [0.05, 0.20]	0.10 [0.05, 0.20]	0.523
Outcomes						
CRRT (%)	64 (29.0)	372 (26.1)	0.42	63 (29.3)	54 (25.1)	0.386
Duration of CRRT (d), median (IQR)	1.82 [0.60, 4.34]	1.80 [0.76, 4.02]	0.726	1.86 [0.65, 4.54]	2.28 [0.85, 5.00]	0.144
Duration of ventilation (d), median (IQR)	2.33 [0.83, 4.28]	1.83 [0.79, 3.82]	0.153	2.33 [0.81, 4.30]	2.16 [0.78, 4.16]	0.768
LOS ICU (d), median (IQR)	9.74 [6.72, 14.84]	7.56 [3.94, 13.41]	<0.001	9.71 [6.70, 14.54]	9.10 [5.14, 14.82]	0.322
LOS hospital (d), median (IQR)	18.82 [11.02, 27.12]	13.79 [7.36, 22.63]	<0.001	18.75 [10.94, 27.02]	15.00 [9.64, 23.69]	0.025
28-day mortality (%)	70 (31.7)	639 (44.9)	<0.001	70 (32.6)	87 (40.5)	0.109
In-hospital mortality within 28 days (%)	60 (27.1)	586 (41.2)	<0.001	60 (27.9)	78 (36.3)	0.079
90-day mortality (%)	90 (40.7)	777 (54.6)	<0.001	90 (41.9)	112 (52.1)	0.042
In-hospital mortality within 90 days (%)	65 (29.4)	652 (45.8)	<0.001	65 (30.2)	92 (42.8)	0.009

DEX, dexmedetomidine; AKI, acute kidney injury; CCI, Charlson comorbidity index; CRRT, continuous renal replacement treatment; GCS, Glasgow coma scale; IQR, interquartile range; LOS, length of stay; MAP, mean arterial pressure; SAPS II, simplified acute physiology II; SOFA, sequential organ failure assessment; SpO_2_, saturation oxygen of pulse; WBCs, White blood cells; yr, year; HTN: hypertension; ARDS, acute respiratory distress syndrome.

NEq/MAP was compared between the two groups using an independent t-test at six timepoints (0 h, 6 h, 12 h, 24 h, 48 h, and 72 h) after the administration of sedatives. For secondary outcomes including serum creatinine level, daily urine output, heart rate, CO, CPOT, RASS, ALT level, AST level, TBIL level, PaO_2_/FiO_2_, and lactate level, group comparisons were performed using chi-square or t-test at four timepoints (0 h, 24 h, 48 h, and 72 h) after sedative administration. All continuous values at each time point are described as median ± interquartile range (IQR) and *p*-values were corrected using the Bonferroni method (threshold $\alpha^{\prime }$=0.05/the number of timepoints).

A generalized linear mixed model (GLMM) was used to assess longitudinal changes in outcomes through the main effects of DEX and time. The confounding factors were adjusted in the GLMM including the baseline demographic and clinical parameters in Table 1, including age, sex, ethnicity, marital status, insurance, admission type, weight, respiratory rate, heart rate, temperature, MAP, SpO_2_, WBCs, platelets, lactate, serum creatinine, SAPS II, SOFA, CCI, GCS score, urinary output within 24 h, PaO_2_/FiO_2_, infection sites, AKI stage, the severity of ARDS, surgery, hypertension, anti-HTN treatment, analgesic drugs including fentanyl and morphine, doses of NEq, CPOT, and RASS. A value of *p* (two-sided) of 0.05 was the indicator of statistical significance.

SAS (9.4) for Windows was used for all analyses.

## Results

### Characteristics of the patients

**Figure 1:**
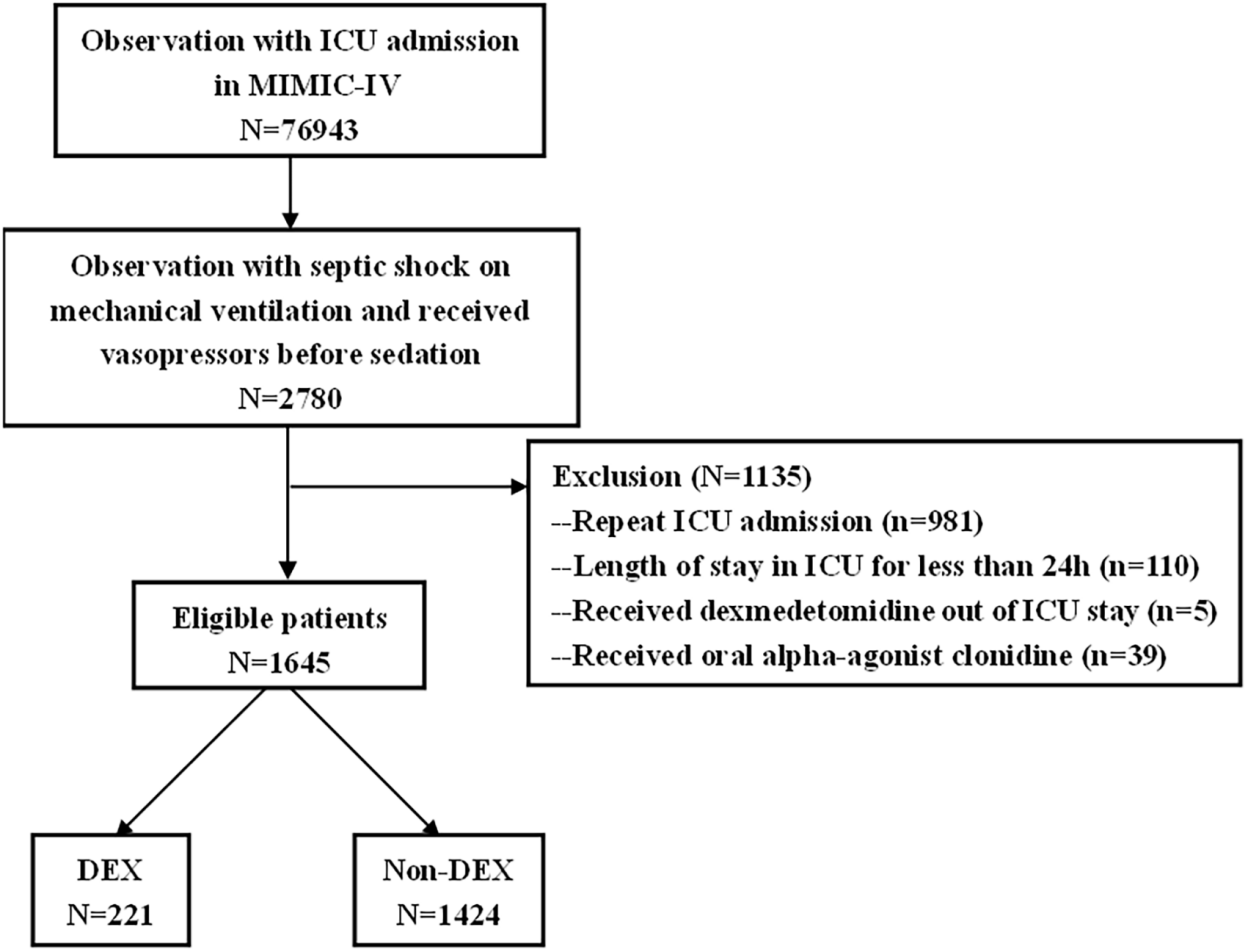
Study flowchart of eligible patients in MIMIC-IV database.

There were 76,943 patients with ICU admission records (Figure 1). After applying our inclusion and exclusion criteria, 430 patients were eligible for the analysis after PSM. The baseline characteristics of DEX and non-DEX groups before and after PSM are shown in Table 1. The baseline PaO_2_/FiO_2_ and proportion of patients with ARDS between the two groups and the percentage of ARDS patients who received DEX or non-DEX were similar in our study. In addition, patients in DEX group had decreased SAPS II scores and lower NE doses and lactate level than those in non-DEX group before PSM while no significant difference was observed after PSM. There was no statistical difference between the two groups in the proportion of patients with different degrees of ARDS and AKI, and the proportion of patients receiving CRRT, baseline serum creatinine, and urinary output within the first 24 h of the two groups was also similar after PSM. The DEX group had a similar proportion of patients with different CPOT and a higher proportion of patients with RASS scores between 1 and 4 points at 24 h, 48 h, and 72 h (Tables 2, 3).

**Table 2:** The critical care pain observation tool (CPOT) at the timepoint of 24 h, 48 h, and 72 h after DEX/non-DEX administration.

Time after DEX/ non-DEX sedation, h	CPOT	Total *n* (%)	DEX *n* (%)	Non-DEX *n* (%)	*p*
24	0	30 (34.1)	20 (34.5)	10 (33.3)	0.221
	1–3	33 (37.5)	18 (31.0)	15 (50.0)	
	4–6	22 (25.0)	17 (29.3)	5 (16.7)	
	7–10	3 (3.4)	3 (5.2)	0 (0.0)	
48	0	19 (27.9)	13 (27.7)	6 (28.6)	0.240
	1–3	29 (42.6)	17 (36.2)	12 (57.1)	
	4–6	15 (22.1)	12 (25.5)	3 (14.3)	
	7–10	5 (7.4)	5 (10.6)	0 (0.0)	
72	0	17 (32.7)	10 (29.4)	7 (38.9)	0.713
	1–3	18 (34.6)	11 (32.4)	7 (38.9)	
	4–6	15 (28.8)	11 (32.4)	4 (22.2)	
	7–10	2 (3.8)	2 (5.9)	0 (0.0)	

**Table 3:** The Richmond agitation sedation scale (RASS) at the timepoint of 24 h, 48 h, and 72 h after DEX/non-DEX administration.

Time after DEX/ non-DEX sedation, h	RASS	Total *n* (%)	DEX *n* (%)	Non-DEX *n* (%)	*p*
24	1−4	99(25.0)	63(34.2)	36(17.0)	<0.001
	−2-0	151(38.1)	76(41.3)	75(35.4)	
	−3-(−5)	146(36.9)	45(24.5)	101(47.6)	
48	1–4	72(19.8)	50(30.3)	22(11.1)	<0.001
	−2-0	156(42.9)	68(41.2)	88(44.2)	
	−3-(−5)	136(37.4)	47(28.5)	89(44.7)	
72	1–4	58(17.8)	42(27.1)	16(9.4)	<0.001
	−2-0	164(50.3)	78(50.3)	86(50.3)	
	−3-(−5)	104(31.9)	35(22.6)	69(40.4)	

### DEX administration was associated with decreased NEq/MAP

**Figure 2:**
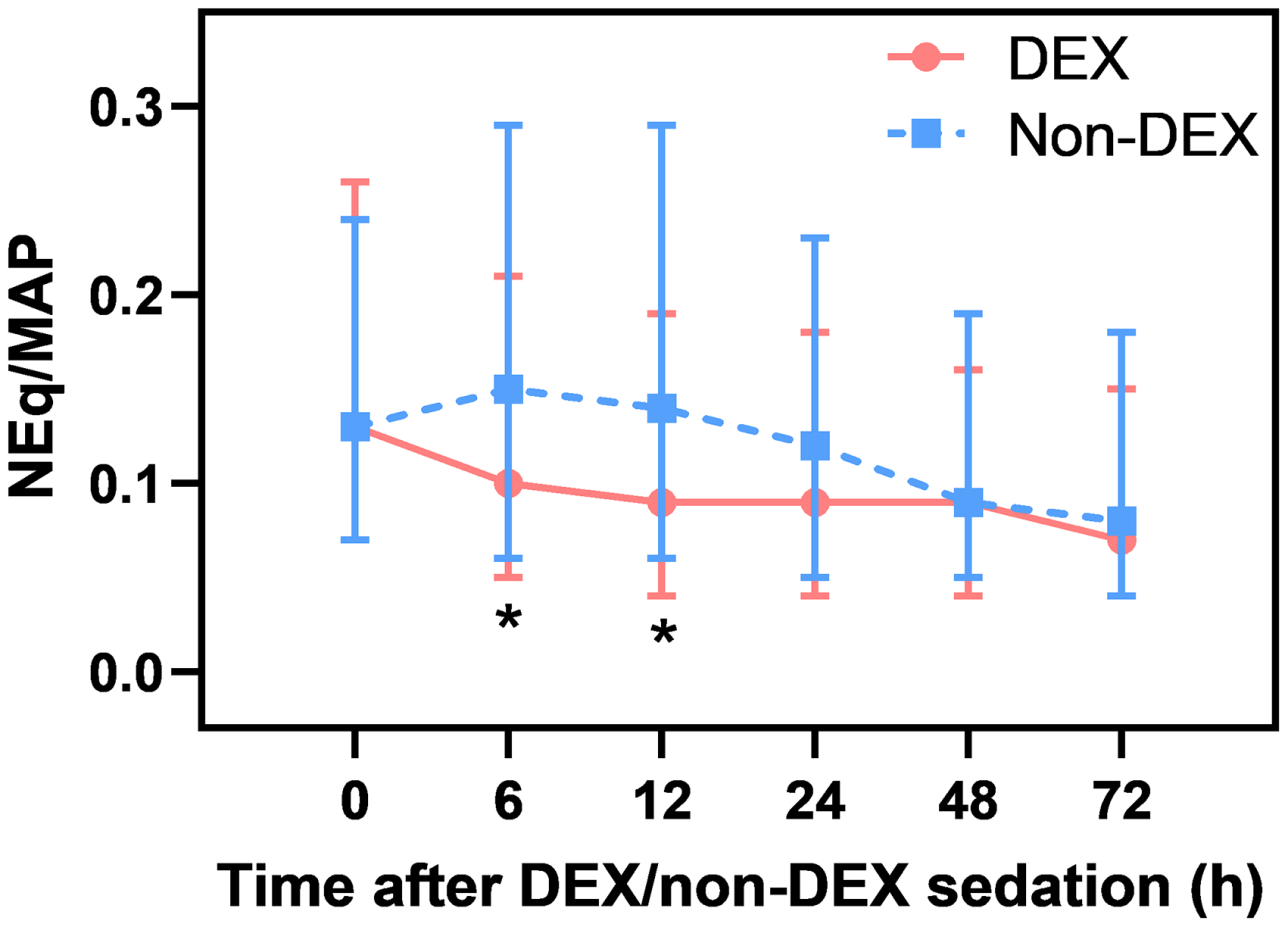
The effect of DEX/non-DEX administration on NEq/MAP (*p* < 0.008).

After Bonferroni correction, a significant difference in NEq/MAP was found between the DEX and non-DEX groups at 6 h and 12 h after sedation (all *p*<$\alpha^{\prime }=$0.008, the threshold for Bonferroni correction was 0.05/6) (Figure 2; Supplementary Table 1). In order to avoid the influence of different levels of sedation and analgesia on the dose of vasopressor drugs, we regarded the level of sedation and analgesia (CPOT, RASS) between the two groups as confounders and adjusted them in our GLMM analysis. And the confounders, include different severity of ARDS and AKI, SAPS II score, baseline NE doses, MAP, lactate level, PaO_2_/FiO_2_, and so on (listed in Table 1 with *p* < 0.05) were all adjusted in GLMM analysis. DEX group was also significantly associated with a decreased NEq/MAP ratio in the first 72 h compared to that in the non-DEX group (difference = 0.05, 95% CI = –0.02–0.08, *p* = 0.002) under the GLMM analysis (Table 4).

**Table 4:** Primary and secondary outcomes after generalized linear mixed model (GLMM) analysis.^*^

Outcomes	Estimate	Lower 95% CI	Upper 95% CI	*p*
NEq/MAP, ug/kg/min	0.05	−0.02	0.08	0.002
	OR			
28-day mortality	0.62	0.38	1.02	0.061
90-day mortality	0.60	0.37	0.94	0.030

*Generalized linear mixed models (GLMM) adjusted for confounders, including age, sex, ethnicity, marital status, insurance, admission type, weight, respiratory rate, heart rate, temperature, MAP, SpO_2_, WBC, Platelets, lactate, serum creatinine, SAPS II, SOFA, CCI, GCS score, urinary out within 24 h, PaO_2_/FiO_2_, infection sites, AKI stage, severity of ARDS, surgery, hypertension, antihypertensive treatment, analgesia drugs including fentanyl, and morphine, doses of NEq, listed in Table 1 and CPOT and RASS.

When compared with the timepoint of 0 h, DEX-only sedation showed a tendency of slightly decreased NEq/MAP ratio at 6 h, 12 h, 24 h, and 72 h and a significant association with reduced NEq/MAP at 48 h (*p* < 0.05). However, non-DEX sedation showed a higher NEq/MAP ratio at 6 h and 12 h than at 0 h (Supplementary Figures 1A,B).

### Other hemodynamic parameters in DEX and non-DEX group

**Figure 3:**
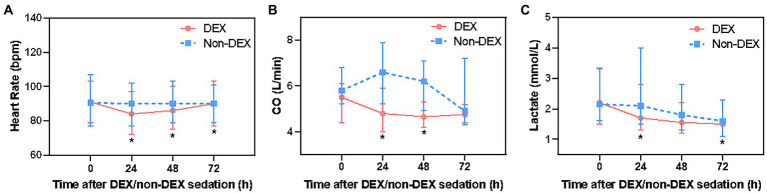
The effect of DEX/non-DEX administration on other hemodynamic parameters in patients with septic shock and mechanical ventilation. **(A)** Heart rate. **(B)** CO. **(C)** Lactate level. *Statistically significant after Bonferroni correction (*p* < 0.0125).

After Bonferroni correction, DEX only administration was associated with a decrease in heart rate of septic shock patients at 24 h, 48 h, and 72 h (*p* < 0.0125, the threshold for Bonferroni correction was 0.05/4 = 0.0125) when compared with non-DEX group (Figure 3A; Supplementary Table 1). The DEX group also showed an association with decreased CO at 24 h and 48 h (p < 0.0125) (Figure 3B; Supplementary Table 1). In terms of tissue perfusion and cellular metabolism, there was a significant difference in lactate level between the groups at the timepoint of 24 h and 72 h (all *p* < 0.0125) (Figure 3C; Supplementary Table 1).

Compared with the timepoint of 0 h within DEX group, heart rate at 24 h was significantly lower, but gradually recovered at 72 h. There was a mild reduction in CO at 24 h, 48 h, and 72 h. Within non-DEX group, heart rate and CO in DEX group were similar at 24 h, 48 h, and 72 h with no significant difference when compared with 0 h (Supplementary Figures 1C–F).

### DEX administration was associated with improved AST and PaO_2_/FiO_2_ level

**Figure 4:**
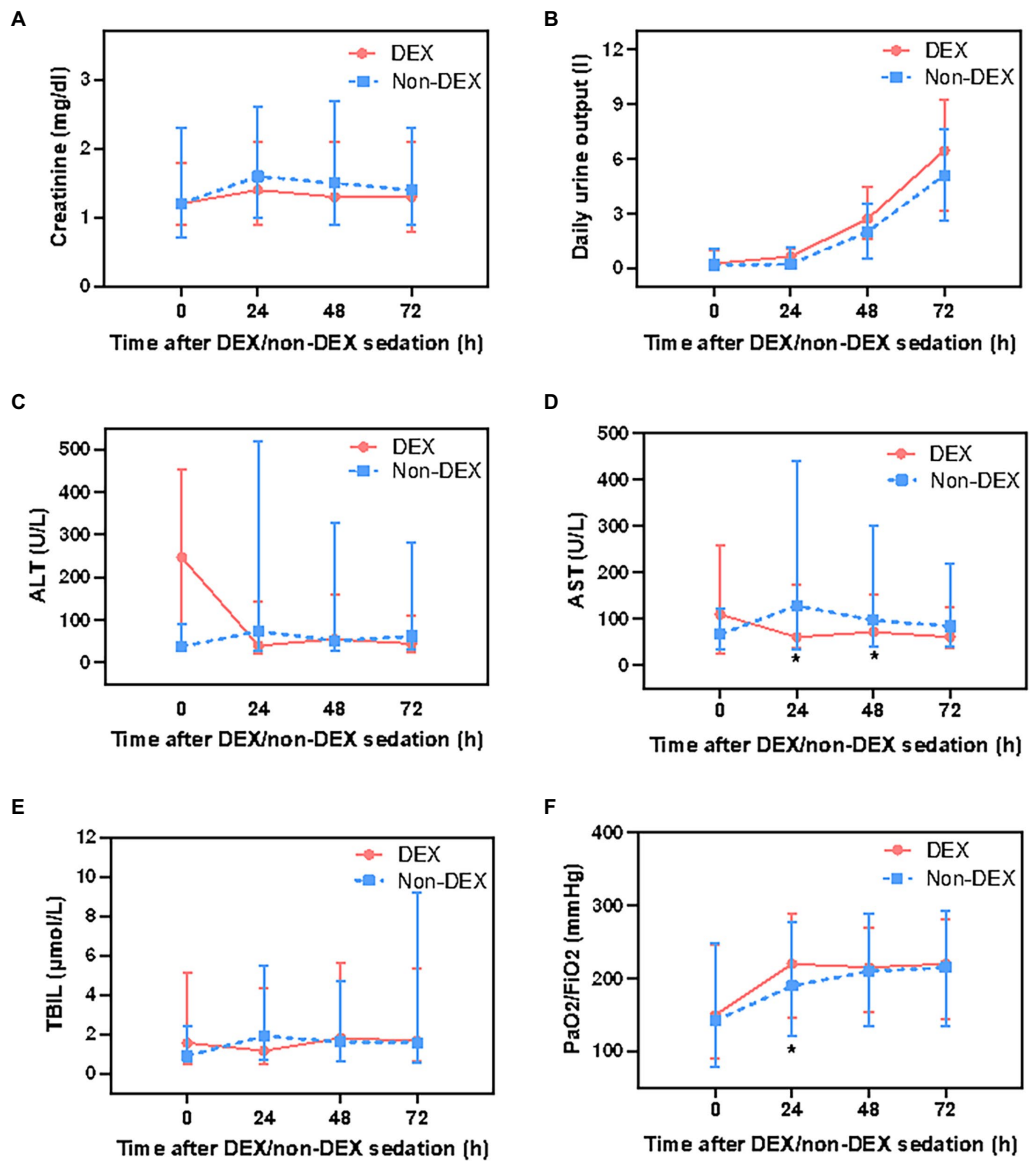
The effect of DEX/non-DEX administration on parameters of multiple organ functions in patients with septic shock and mechanical ventilation. **(A)** Serum creatinine level. **(B)** Daily urine output. **(C)** ALT level. **(D)** AST level. **(E)** TBIL level. **(F)** PaO_2_/FiO_2_. *Statistically significant after Bonferroni correction (*p* < 0.0125).

In assessing the biochemical markers of the kidney, residual renal function was not recorded in the database, therefore, we only analyzed serum creatinine and daily urinary output. DEX group had slightly lower serum creatinine level and more daily urine output at 24 h, 48 h, and 72 h than that of the non-DEX group (all *p* > 0.0125, the threshold for Bonferroni correction was 0.05/4 = 0.0125) (Figures 4A,B; Supplementary Table 1).

The level of AST was significantly lower in DEX group than in non-DEX group at 24 h and 48 h (all *p* < 0.0125) whereas the levels of ALT and TBIL were slightly lower at 24 h in DEX group with no statistical significance (all *p* > 0.0125). In addition, when compared with 0 h, AST, ALT, and TBIL levels decreased at 24 h after DEX-only sedation (Figures 4C–E; Supplementary Table 1). Regarding the biochemical markers of lung, patients in DEX group had higher PaO_2_/FiO_2_ ratio than that in non-DEX group at 24 h (*p* < 0.0125) (Figure 4F; Supplementary Table 1).

### DEX administration was significantly associated with reduced 90-day mortality

Univariate analysis after PSM showed that DEX group was associated with decreased 90-day mortality (41.9 vs. 52.1%, *p* = 0.042) and in-hospital mortality within 90 days (30.2 vs. 42.8%, *p* = 0.009), with a significantly longer hospital length of stay (LOS) (18.75 vs. 15.00 d, *p* = 0.025) (Table 1). After GLMM analysis, DEX group was also associated with reduced 90-day mortality (OR = 0.60, 95% CI = 0.37–0.94, *p* = 0.030) compared to non-DEX group (Table 4). To further explore the difference in hospital and ICU LOS of patients who survived within 90 days, we made an inter group comparison among survivors in the two groups. We found that survivors in DEX group, which had a lower 90-day mortality, also had a longer LOS in ICU and hospital, although there was no statistical difference (Table 5). However, DEX group was not associated with reduced 28-day mortality (OR = 0.62, 95% CI = 0.38–1.02, *p* = 0.061) (Table 4).

**Table 5:** Comparison of LOS ICU and hospital of survivors within 90 days in DEX and non-DEX group.

	DEX	Non-DEX	*p*
LOS ICU, d, median (IQR)	9.62 [6.23, 13.40]	8.79 [5.70, 14.82]	0.423
LOS hospital, d, median (IQR)	20.31 [12.85, 30.06]	18.01 [12.72, 25.81]	0.984

## Discussion

In this retrospective study, we found that DEX-only regimen may be associated with decreased vasopressor requirements, improved AST and PaO_2_/FiO_2_ levels, and reduced 90-day mortality in patients with septic shock. Our study highlights the hemodynamic advantage of dexmedetomidine for as long as 72 h after drug exposure, which suggests that DEX might be preferred for septic shock treatment.

Current literature suggests that DEX administration could lower vasopressor requirements in patients with septic shock (15–17). Mechanistically, some animal studies speculated that DEX’s sympatholytic effect (25) reduced plasma catecholamine levels in a time-dependent (26, 27) and dose-dependent manner (28) through its highly selective α2-adrenergic receptor agonist function (29), and patients with refractory shock usually show excessive catecholamine release (7). From this perspective, DEX may help reduce the resistance of vascular smooth muscle cells to catecholamines, thereby reducing the demand for vasopressor drugs. In addition, a study revealed that a low plasma concentration of DEX acts on α2A receptors in vascular smooth muscle cells, leading to vasodilatory effects, whereas high concentrations of DEX would directly activate α2B receptors, which exert vasoconstrictive effects and increase blood pressure (28). To be more objective in explaining vasopressor effects in this retrospective study, we chose NEq/MAP ratio (a higher ratio indicates higher vasopressor requirements to maintain a certain MAP) (15) as our primary endpoint to account for differences in target MAP between DEX and non-DEX groups. After PSM and GLMM analyses with adjustment for MAP and other confounders, our study revealed that there was still an association between DEX and reduction in NEq/MAP in the first 72 h after sedation. Our analysis is based on a large population focusing on longer observation durations, as long as 72 h. Further, we compared different timepoints after DEX/non-DEX sedation. The present study is the first to compare the potential roles of DEX and other sedatives in reducing vasopressor requirements in septic shock patients, and to evaluate the impact on vasopressor needs during sedation based on horizontal comparisons.

Inconsistent with our study, a multicenter randomized trial (DESIRE trial) included 201 sepsis patients requiring mechanical ventilation and found no statistically significant improvement in mortality or ventilator-free days in DEX group, despite the possibility of underpowered mortality (30). Although our study was retrospective, we believe that our study has certain strengths: first, compared to the DESIRE trial, our study focused on vasopressor reactivity (a key physiological effect). Second, the study population of the DESIRE trial included patients with sepsis, and only a subset with septic shock. Our study included patients with septic shock, which decreased the heterogeneity. Third, our study analyzed various hemodynamic parameters and organ biochemical markers at different time points during the first 72 h of shock. Fourth, to avoid the influence of other sedatives, we included DEX-only versus other sedatives to more directly and objectively reflect the impact of DEX on vasopressor demand. Our previous study revealed that DEX administration showed no significant difference in vasopressor requirements in patients with sepsis associated AKI (SA-AKI) (31), which was inconsistent with the results of the present study and might be due to the different study populations of the two studies. Our study focused on septic shock patients with mechanical ventilation, whereas Hu et al. (31) included patients with SA-SKI.

Several factors may contribute to elevated HR during septic shock, including hypovolemia and septic cardiomyopathy (32, 33). Studies have shown that treatments that reduce HR in septic shock significantly improve outcomes (29). DEX has been found to reduce HR, likely through its sympatholytic properties (25). In studies investigating the effects of DEX in healthy individuals, DEX was found to decrease CO, presumably by decreasing the heart rate (28). In our study, we revealed that both the heart rate and CO decreased at 24 h, but as time passed, the heart rate gradually recovered to the baseline level and CO tended to be stable at 72 h, which indicated that DEX might have hemodynamic advantages in septic shock patients.

DEX has been confirmed to have various organ protective roles in several animal studies (34–40). However, whether DEX has multiple organ-protective effects in patients with septic shock is unknown. In this study, we found that DEX might be related to improvements in AST and PaO_2_/FiO_2_ levels. Since the parameters that we selected in the study only partly reflected organ functions, other parameters, such as residual renal function, were not recorded in the database. Further clinical trials are needed to confirm the relationship between DEX and multi-organ functions. Unexpectedly, we showed that DEX-only sedation was associated with a longer length of hospital stay, and a lower 90-day mortality rate in septic shock patients with mechanical ventilation. One of the reasons for this might be that the longer survival time in DEX group indicated that patients would be hospitalized longer. That is, patients in DEX group had both lower 90-day mortality and in-hospital mortality within 90 days in univariate analysis after PSM, the lower in-hospital mortality within 90 days meant that patients in DEX group had longer hospitalizations, which contributed to the longer LOS in hospital. In addition, further analysis of survivors in the two groups also revealed that survivors in DEX group, which had a lower 90-day mortality, had a longer LOS in ICU and hospital, although the difference was not statistically significant. As for 28-day mortality and 28-day in hospital mortality, a similar tendency was observed, but without a significant difference. Similarly, a study (41) showed improved patient outcomes such as reduced mortality, and less delirium/coma. The possible benefits of DEX may be related to its intrinsic α2-adrenergic receptor agonist characteristics and reduced moderate inflammatory reactions and the effects of DEX sedation and auxiliary analgesia (42–44). However, the specific mechanisms need to be studied further.

The limitations of this study originate from its retrospective nature, there might be measurement bias because of the long time period, which ranged from 2008 to 2019, although PSM analysis was applied to reduce selection bias. The results of this study only showed a statistical association between DEX and reduced vasopressor requirements, which requires further randomized controlled trials to confirm this. The costs of DEX/non-DEX group were not recorded, and specific DEX doses used in each patient were not explored in the present study, thus we were unable to analyze the economic benefits and any dose-dependent effects of DEX. Additionally, with progress in the treatment of septic shock, many other advances, such as how to use the ventilators, fluids, or nutrition management (45), would also influence the correlations between DEX and vasopressor requirements. Moreover, some small clinical trials have demonstrated that the association between DEX and reduced vasopressor requirements was evidenced in patients with more severe sepsis or refractory septic shock (16, 17, 30), and we only included patients with septic shock and failed to distinguish refractory septic shock from them. Therefore, conclusions regarding the beneficial effects of DEX should be made cautiously.

## Conclusion

Among septic shock patients on mechanical ventilation, DEX may be associated with decreased vasopressor requirements, improved AST and PaO_2_/FiO_2_ levels, and reduced 90-day mortality in septic shock patients up to 72 h after drug exposure.

The use of DEX in septic shock patients on mechanical ventilation in critical care settings warrants further study.

## Data availability statement

The original contributions presented in the study are included in the article/Supplementary material, further inquiries can be directed to the corresponding authors.

## Ethics statement

Ethical review and approval was not required for the study on human participants in accordance with the local legislation and institutional requirements. Written informed consent for participation was not required for this study in accordance with the national legislation and the institutional requirements.

## Author contributions

LL helped to analyze the data and wrote the manuscript. XS analyzed datasets. MX helped to reviewed and modify the manuscript. KK helped to modify the manuscript. ZC helped to design the work. SZ extracted data from the database. ZZ helped to write the manuscript. SA analyzed the data and reviewed the data result. BX designed the work and reviewed the manuscript. All authors read and approved the final manuscript.

## Funding

This work was supported by the Clinical Research Program of NanFang Hospital, Southern Medical University [grant number 2018CR047]. The National Natural Science Foundation of China [Grant number 62076253] and the Natural Science Foundation of Guangdong Province [Grant number 2021A1515010077].

## Conflict of interest

The authors declare that the research was conducted in the absence of any commercial or financial relationships that could be construed as a potential conflict of interest.

## Publisher’s note

All claims expressed in this article are solely those of the authors and do not necessarily represent those of their affiliated organizations, or those of the publisher, the editors and the reviewers. Any product that may be evaluated in this article, or claim that may be made by its manufacturer, is not guaranteed or endorsed by the publisher.
