# Supplementary material for: Dexmedetomidine only regimen for long-term sedation is associated with reduced vasopressor requirements in septic shock patients: A retrospective cohort study from MIMIC-IV database
Source: Front Med (Lausanne). 2023 Feb 27;10:1107251. doi: 10.3389/fmed.2023.1107251 (PMC10010261; doi:10.3389/fmed.2023.1107251)
Supplement: Supplementary file 1 [file Data_Sheet_1.docx]

Supplementary Material

Dexmedetomidine Only Regimen for Long-Term Sedation is Associated with Reduced Vasopressor Requirements in Septic Shock Patients: A Retrospective Cohort Study from MIMIC-IV Database

Lulan Li^1,2†^, Xiaotong Shi^3†^, Ming Xiong^4^, Karen Kong^4^, Zhongqing Chen^2^, Shiyu Zhou^3^, Zhenhua Zeng^2^, Shengli An^3*^, Bo Xu^1*^

*** Correspondence:**

Shengli An

Email: asl0418@126.com

Bo Xu

Email: [xubo333@hotmail.com](mailto:xubo333@hotmail.com)

# Supplementary Table

**STable1** Original data of hemodynamic effects and parameters of different organs in DEX and non-DEX group.

|  | DEX | Non-DEX | p |
| --- | --- | --- | --- |
| NEq/MAP (μg/kg/min), median (IQR) |  |  |  |
| 0h | 0.13 [0.07, 0.26] | 0.13 [0.07, 0.24] | 0.798 |
| 6 h | 0.10 [0.05, 0.21] | 0.15 [0.06, 0.29] | <0.001 |
| 12 h | 0.09 [0.04, 0.19] | 0.14 [0.06, 0.29] | <0.001 |
| 24 h | 0.09 [0.04, 0.18] | 0.12 [0.05, 0.23] | 0.045 |
| 48 h | 0.09 [0.04, 0.16] | 0.09 [0.05, 0.19] | 0.098 |
| 72 h | 0.07 [0.04, 0.15] | 0.08 [0.04, 0.18] | 0.606 |
| Heart rate (bpm), median (IQR) |  |  |  |
| 0 h | 91.00 [79.00, 103.00] | 90.50 [77.00, 107.00] | 0.917 |
| 24 h | 84.00 [72.00, 97.00] | 90.00 [77.00, 102.00] | <0.001 |
| 48 h | 86.00 [75.00, 101.00] | 90.00 [79.00, 103.00] | 0.001 |
| 72 h | 90.00 [77.00, 103.00] | 90.00 [79.00, 101.00] | <0.001 |
| CO (L/min), median (IQR) |  |  |  |
| 0 h | 5.50 [4.40, 6.10] | 5.80 [5.20, 6.80] | 0.296 |
| 24 h | 4.80 [4.00, 5.90] | 6.60 [5.20, 7.90] | <0.001 |
| 48 h | 4.65 [4.20, 5.30] | 6.20 [4.93, 7.10] | <0.001 |
| 72 h | 4.75 [4.40, 5.18] | 4.90 [4.30, 7.20] | 0.975 |
| Lactate (mg/dL), median (IQR) |  |  |  |
| 0 h | 2.20 [1.49, 3.31] | 2.15 [1.60, 3.33] | 0.876 |
| 24 h | 1.70 [1.30, 2.80] | 2.10 [1.50, 4.00] | <0.001 |
| 48 h | 1.60 [1.20, 2.20] | 1.80 [1.30, 2.80] | 0.013 |
| 72 h | 1.60 [1.10, 2.30] | 1.80 [1.20, 3.20] | 0.011 |
| Serum creatinine (mg/dL), median (IQR) |  |  |  |
| 0 h | 1.20 [0.90, 1.80] | 1.20 [0.70, 2.30] | 0.82 |
| 24 h | 1.40 [0.90, 2.10] | 1.60 [1.00, 2.60] | 0.021 |
| 48 h | 1.30 [0.90, 2.10] | 1.50 [0.90, 2.70] | 0.090 |
| 72 h | 1.30 [0.80, 2.10] | 1.40 [0.90, 2.30] | 0.487 |
| Daily urine output (L), median (IQR) |  |  |  |
| 0 h | 0.26 [0.05, 0.96] | 0.19 [0.05, 1.09] | 0.545 |
| 24 h | 0.65 [0.05, 1.09] | 0.23 [0.04, 1.17] | 0.769 |
| 48 h | 2.71 [1.61, 4.46] | 2.01 [0.57, 3.51] | 0.049 |
| 72 h | 6.46 [3.18, 9.22] | 5.11 [2.61, 7.63] | 0.049 |
| ALT (U/L), median (IQR) |  |  |  |
| 0 h | 247.00 [40.00, 454.00] | 37.50 [29.00, 89.00] | 0.501 |
| 24 h | 40.00 [20.00, 143.00] | 74.00 [27.25, 519.50] | 0.075 |
| 48 h | 55.00 [27.75, 161.25] | 51.00 [29.00, 327.00] | 0.031 |
| 72 h | 44.00 [23.00, 108.50] | 62.00 [30.75, 281.25] | 0.04 |
| AST (U/L), median (IQR) |  |  |  |
| 0 h | 109.50 [24.75, 359.25] | 67.00 [35.00, 123.00] | 0.582 |
| 24 h | 61.00 [36.50, 172.50] | 129.00 [36.00, 441.00] | 0.003 |
| 48 h | 72.00 [42.00, 151.75] | 97.50 [40.00, 302.00] | 0.002 |
| 72 h | 61.00 [36.50, 126.00] | 84.50 [39.50, 220.00] | 0.011 |
| TBIL (mg/dL), median (IQR) |  |  |  |
| 0 h | 1.55 [0.50, 5.15] | 0.90 [0.60, 2.40] | 0.563 |
| 24 h | 1.15 [0.50, 4.35] | 1.90 [0.70, 5.50] | 0.338 |
| 48 h | 1.80 [0.60, 5.65] | 1.60 [0.60, 4.70] | 0.506 |
| 72 h | 1.65 [0.60, 5.35] | 1.55 [0.58, 9.17] | 0.196 |
| PaO_2_/FiO_2_ (mmHg), median (IQR) |  |  |  |
| 0 h | 150.00 [91.00, 245.83] | 142.00 [78.00, 248.57] | 0.610 |
| 24 h | 220.00 [146.00, 290.00] | 190.00 [121.35, 277.23] | <0.001 |
| 48 h | 215.50 [153.50, 270.00] | 210.00 [134.17, 288.17] | 0.897 |
| 72 h | 220.00 [145.00, 281.88] | 215.00 [135.50, 292.50] | 0.931 |

# Supplementary Figure


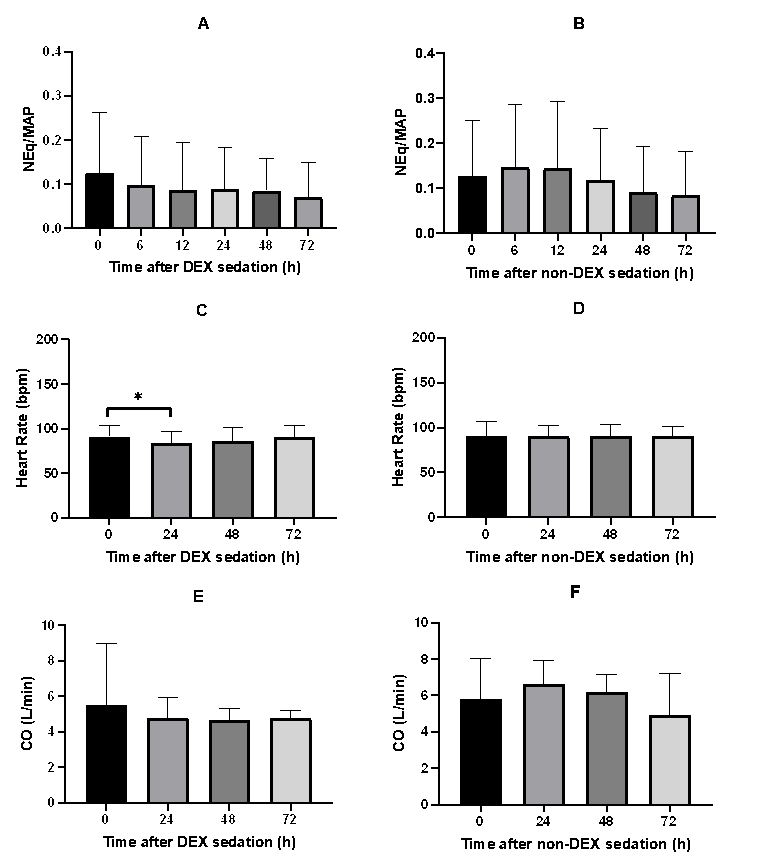


**SFigure 1** Comparison of each timepoint of the hemodynamic effects in DEX/non-DEX group.
